# Evaluating the Aphrodisiac Potential of *Mirabilis jalapa* L. Root Extract: Phytochemical Profiling and In Silico, In Vitro, and In Vivo Assessments in Normal Male Rats

**DOI:** 10.3390/molecules28176314

**Published:** 2023-08-29

**Authors:** Asad Ur Rahman, Fiaz Alam, Muhammad Khan, Muhammad Sarfraz, Abdul Basit, Tawseef Ahmad, Muhammad Ali Khokhar, Sayyad Ali, Kifayat Ullah Khan

**Affiliations:** 1 Department of Pharmacognosy and Pharmaceutical Botany, Faculty of Pharmaceutical Sciences, Prince of Songkla University, Hat-Yai 90112, Thailand; asadrahman359@gmail.com (A.U.R.); mali.khokhar@usindh.edu.pk (M.A.K.); 2 Department of Pharmacy, COMSATS University Islamabad, Abbottabad Campus, Abbottabad 22060, Pakistan; sayad.ali6@gmail.com; 3 Department of Pharmacology, Federal Urdu University of Arts, Science and Technology, Karachi 75300, Pakistan; 4 College of Pharmacy, Al Ain University, Al Ain 64141, United Arab Emirates; muhammad.sarfraz@aau.ac.ae; 5 Department of Pharmaceutical Chemistry, Faculty of Pharmaceutical Sciences, Prince of Songkla University, Hat-Yai 90112, Thailand; 6 Department of Clinical Pharmacy, Faculty of Pharmaceutical Sciences, Prince of Songkla University, Hat-Yai 90112, Thailand; tausifsafi95@gmail.com; 7 Department of Pharmacology, Faculty of Pharmacy, University of Sindh, Jamshoro 71000, Pakistan; 8 Quaid-e-Azam College of Pharmacy, Sahiwal 54000, Pakistan; kifayat.rph@yahoo.com

**Keywords:** erectile dysfunction, GC-MS, in vivo assay, *Mirabilis jalapa* L., molecular docking, phosphodiesterase-5, phytochemical, root extract

## Abstract

The traditional use of *Mirabilis jalapa* L. roots to enhance male sexual performance prompted us to assess the in silico, in vitro, and in vivo aphrodisiac activities of its hydroethanolic extract using normal male rats. Spectroscopic characterization indicated the presence of ß-D-glucopyranoside, methyl-1,9-benzyl-2,6-dichloro-9H-purine, and Bis-(2-ethylhexyl)-phthalate; these compounds have a significant inhibitory effect on the phosphodiesterase-5 (PDE-5) enzyme in silico evaluation and minerals (including zinc, cadmium, and magnesium). Other phytochemical analyses revealed the presence of phenolic compounds and flavonoids. These phytochemicals and minerals may contribute to the aphrodisiac activities of the extract. Additionally, the in vivo study revealed that the administration of *M. jalapa* root extract (300 mg/kg) significantly enhanced (*p* < 0.01, *p* < 0.03) mount, intromission, and ejaculation frequencies while significantly (*p* < 0.05) decreasing the mount and intromission latencies, as well as the post-ejaculatory interval time, in comparison with the standard drugs sildenafil and ginseng, resulting in enhanced erection and sexual performance in the rats. Furthermore, the extract significantly (*p* < 0.05) increased penile reflexes and also elevated the levels of testosterone and luteinizing hormones. Extract (300 mg/kg) significantly (*p* < 0.05) inhibited the PDE-5 enzyme in an in vitro study. Concludingly, the comprehensive findings of this study suggest that a standardized herbal extract derived from *M. jalapa* roots alleviates erectile dysfunction and premature ejaculation in male rats. *M. jalapa* root extract proved to be an alternative treatment for erectile dysfunction and premature ejaculation.

## 1. Introduction

Erectile dysfunction (ED) is a medical condition characterized by an inability to achieve or maintain a penile erection firm enough to fulfill satisfactory sexual intercourse. It can be a temporary issue or can persist over an extended period [1]. ED is a prevalent clinical condition that predominantly impacts men over the age of 40. Alongside the traditional causes of erectile dysfunction, such as diabetes mellitus and hypertension, various common lifestyle elements like obesity, inadequate physical activity, and lower urinary tract symptoms have also been associated with the emergence of this condition [2]. ED deeply impacts one’s quality of life, often diminishing self-esteem and leading to emotional distress like depression or anxiety. This condition can strain relationships by reducing intimacy and creating communication barriers. Socially, affected individuals might isolate themselves or avoid forming new relationships [3]. The worldwide prevalence of ED varies across different studies and populations. However, estimates suggest that ED affects a significant number of men globally. According to research, it is estimated that by 2025, approximately one-third of a billion men across the globe will be inflicted by ED [4].

A range of conventional medications are available in the market to treat or manage ED. Among them, sildenafil citrate (popularly recognized as Viagra), vitamin E, levodopa (a dopamine precursor), and amyl nitrite stand out as frequently utilized therapeutic options. These medications target various mechanisms underlying sexual dysfunction and are prescribed based on individual needs and health conditions [5,6]. However, the primary treatment approach for ED often involves the use of phosphodiesterase-5 inhibitors (PDE-5 inhibitors). Medications like sildenafil and vardenafil, belonging to this class of drugs, are commonly prescribed. PDE-5 inhibitors work by increasing blood flow to the penis, helping to achieve and maintain an optimum erection. Sildenafil has a structural similarity to cyclic guanosine monophosphate (CGMP), and it prevents the phosphodiesterase type 5 (PDE-5) enzyme from breaking down cyclic cGMP by bonding to it competitively. This complex mechanism promotes blood circulation and vasodilation, which in turn promote penile erection [7]. All these conventional medications to treat ED are associated with a plethora of problems including limited efficacy, frequent side effects, development of tolerance on prolonged consumption, possible deterioration of an underlying disease, psychogenic factors, and partner issues. PDE-5 inhibitors like sildenafil might cause headaches, flushing, prolonged erections or vision and hearing loss. In addition, alprostadil can lead to penile pain, urethral burning, or prolonged erections, while testosterone replacement can result in acne, fluid retention, prostate issues, blood clots, etc. [8].

The aforementioned limitations of the conventional drugs employed for the treatment of ED have highlighted the need for novel plant-based entities demonstrating improved efficacy, safety, and affordability. Recently, various phytochemicals have delivered remarkable pharmacological potential to cure a range of ailments due to their enhanced efficacy, non-toxic nature, and little to no side effects [9]. Natural aphrodisiac moieties, derived from plants, animals, or minerals, are believed to exhibit capabilities that can improve sexual desire, performance, and overall sexual health [10]. Indeed, several traditional herbs have been used worldwide to treat ED and are reported to have potential benefits in improving sexual performance. Some of these herbs including *Curculigo orchioides*, *Chlorophytum borivilianum*, *Tribulus terrestris*, *Sida cardifolia*, *Asphaltum*, *Withania somnifera*, *Hibiscus abelmoschus*, *Anacyclus pyrethrum*, and *Asparagus racemosus* have been traditionally recognized for their aphrodisiac properties and have been explored in detail for their marvelous effects on sexual function and libido [11,12,13,14].

*Mirabilis jalapa* L. *(Nyctaginaceae)*, commonly known as the “four o’clock plant”, is a perennial herb cherished globally for its colorful and vibrant blossoms. While it is primarily recognized for its ornamental value, the medicinal benefits of *M. jalapa* cannot be overlooked. Traditional practices from various cultures have harnessed their roots, especially in a powdered form, as a remedy to treat a variety of ailments including erectile dysfunction and premature ejaculation. Recent studies have also highlighted the potential of root extracts from this plant in addressing diabetes mellitus, while also showcasing its noteworthy antioxidant and anti-inflammatory properties [15,16,17]. Additionally, our prior research indicated that the root extract of *M. jalapa* L. significantly enhanced the aphrodisiac responses in male rats with sexually depressed behavior induced by paroxetine [18]. Indeed, the traditional uses and purported medicinal properties of *M. jalapa* L. roots insighted our interest in investigating its potential aphrodisiac properties.

Therefore, our current research presents a comprehensive report on the aphrodisiac capabilities of *M. jalapa* L., specifically utilizing its hydroethanolic root extract. This study adopts a multifaceted approach encompassing phytochemical analysis, in silico predictions, and both in vitro as well as in vivo experiments to thoroughly explore the various facets of the aphrodisiac qualities inherent in the *M. jalapa* root extract. Talking in detail, the current study included a thorough phytochemical analysis of the extract, detailing its phenolic, flavonoid, metal, and mineral compositions. This was followed by a GC-MS assessment to pinpoint the distinct compounds in the root extract. Subsequently, we evaluated the extract’s potential inhibitory effects on the PDE-5 enzyme through in silico and in vitro methods. These preliminary findings were further validated with in vivo tests on healthy male albino rats, comparing the results with standard drugs like sildenafil and ginseng. Notably, this research stands out as the first-ever detailed and comparative study that underscores the aphrodisiac potentials of hydroethanolic extract derived from *M. jalapa* L. roots in normal male albino rats.

## 2. Results and Discussion

Plants are a valuable resource in the advancement of novel medications, attributed to their presumed safety and economic viability. Many herbal remedies have been employed to cure a plethora of ailments including ED. These herbal remedies offer a natural and potentially beneficial approach to addressing ED and have been used traditionally for their therapeutic properties. Their utilization in the treatment of ED highlights the significance of plant-based compounds in the development of alternative treatments for this condition [19]. In our previous study, we demonstrated the significant aphrodisiac potentials of the ethanolic extract of *M. jalapa* roots in male rats with paroxetine-induced ED. However, the current investigation aimed to establish a link between the phytochemical components found in the hydroethanolic extract of *M. jalapa* roots and its potential aphrodisiac properties. In contrast to the previous study, the aphrodisiac model employed in this study involved normal male rats, rather than rats with paroxetine-induced ED. This allowed a broader investigation of the aphrodisiac properties of the *M. jalapa* extracts in a non-induced model.

ED can be attributed to oxidative stress caused by an imbalance between excessive free radicals and antioxidant defenses in the penile cavernous tissues. The presence of an increased number of free radicals contributes to oxidative damage, inflammation, and impairment of nitric oxide signaling pathways, ultimately affecting erectile function. Addressing oxidative stress and maintaining a balance between free radicals and antioxidants is crucial in managing and preventing ED. The hydroethanolic extract derived from the roots of *M. jalapa* was documented to contain an appreciable number of phenolic compounds (358.5 ± 6.00 mg GAE/g of extract) and flavonoids (69.3 ± 3.50 mg quercetin/g of extract). Phenols and flavonoids have been widely recognized for their potent antioxidant activities. These compounds have the ability to scavenge free radicals and reduce oxidative stress, which is known to contribute to the development of erectile dysfunction (ED). By reducing oxidative stress, phenols and flavonoids may help protect the cavernous tissues and maintain normal erectile function [20,21].

In addition to flavonoids and phenols, minerals play a crucial role in the proper functioning of the body, including sexual health. Zinc, in particular, is essential for various aspects of sexual health, such as testosterone production, sperm maintenance, and prostate health. Zinc also contributes to the proper functioning of the immune and digestive systems. Other minerals like calcium, potassium, magnesium, and cadmium are important for maintaining muscle and bone health. Furthermore, these minerals can have a relaxation effect on the corpus cavernosum of the penis, which can positively impact erectile function [22]. In conclusion, an abundant amount of minerals found in the root extract of *M. jalapa* root extract may have definitely contributed to its potential beneficial effects on sexual health. The atomic absorption spectroscopy analysis revealed that a vast amount of zinc, potassium, calcium, magnesium, and cadmium is present in the root extract, as denoted in Table 1**.** In addition to flavonoids, phenols, and minerals, there might be certain other phytochemicals that have aphrodisiac properties.

**Figure 1:**
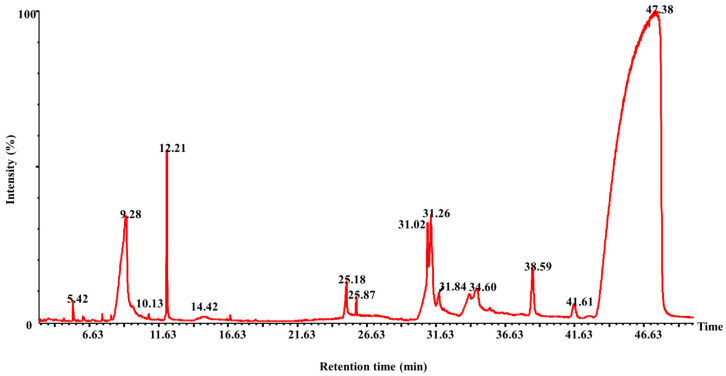
GC-MS Chromatogram of *M. jalapa* root extract.

**Figure 2:**
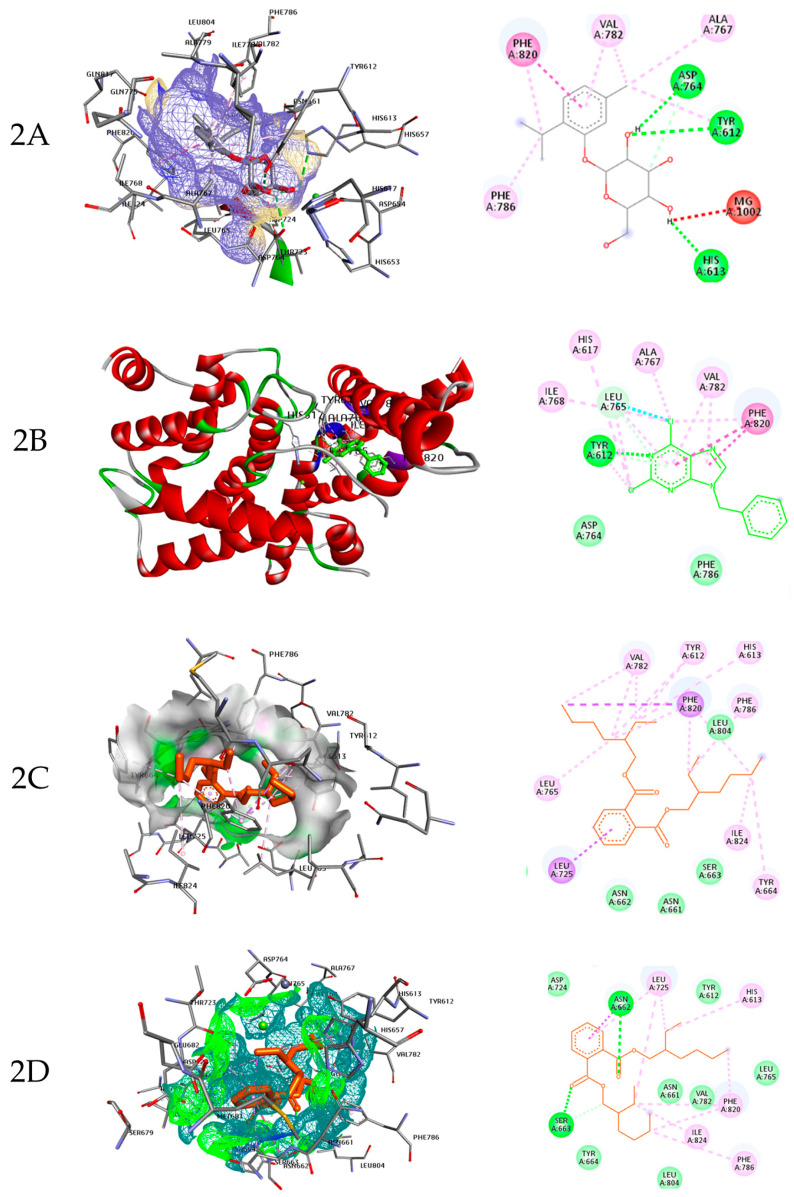
The molecular docking analysis showing the ligand beta-d-glucopyranoside, methyl-1 (**2A**), ligand 9-benzyl-2,6-dichloro-9*H*-purine (**2B**), Bis(2-ethylhexyl) phthalate first ranked (**2C**), and Bis(2-ethylhexyl) phthalate second-ranked pose (**2D**) bonded with the active domain of PDE5. The 3D depicts the docking of ligand to PDE-5. Dashed lines in 2D* depiction show the interaction between the ligand with the different active pockets of the enzyme.

The root extract of *M. jalapa* was further analyzed using the GC-MS technique. The chromatograph revealed several distinct peaks, as shown in Figure 1. Each peak was carefully analyzed by matching it with compounds present in the library, as described in Table 2. This analysis helped in identifying and characterizing the specific compounds present in the root extract, providing valuable information about its chemical constituents. The in silico study involved those phytochemicals that were revealed in the root extract of *M. jalapa* via GC-MS analysis. Among these compounds, beta-d-glucopyranoside, methyl-1,9-benzyl-2,6-dichloro-9H-purine, and Bis(2-ethylhexyl) phthalate exhibited favorable geometric conformations and interactions with the PDE-5 enzyme. The docking data experiment revealed that beta-d-glucopyranoside, methyl-1 exhibited favorable geometric conformations and interactions with the PDE-5 enzyme. The docking data indicated that beta-d-glucopyranoside, methyl-1 displayed inhibitory activity against the PDE-5 enzyme. Inhibition means that these ligands could potentially reduce the enzymatic activity, which is significant for conditions like erectile dysfunction. The PDE-5 enzyme has a catalytic domain containing specific amino acid residues. The residues as Tyr612, His613, Ser661, Thr723, Asp724, Asp764, Leu765, Val782, and Phe786 are vital for the enzyme’s function and interactions. The “active pockets” are regions in the enzyme where ligands can bind and influence its activity. The molecular modeling indicates fundamental interaction among the active pockets and the intended ligand which are crucial for the inhibitory action of the ligand beta-d-glucopyranoside and methyl-1. Molecular modeling interactions show that ligands under study fit exactly to the internal groove of the catalytic domains of PDE-5 where sildenafil links for optimum inhibition activities. Sildenafil is used to treat erectile dysfunction by inhibiting PDE-5, leading to increased levels of cGMP and improved blood flow [23]. Figure 2A, which likely provides visual representations of the docking results for beta-d-glucopyranoside, methyl-1. Figure 2A shows how these compounds bind to the PDE-5 enzymatic catalytic domain.

The ligand 9-benzyl-2,6-dichloro-9H-purine occupies the internal groove of the catalytic domain. It forms bonds that inhibit the enzyme’s catalytic pockets. The interactions are highlighted in Figure 2B as dotted lines, indicating how this ligand inhibits PDE-5. Hydrophobic interactions involving amino acids like Leu725, Val782, and Leu804 which contribute to the overall inhibitory activity of the ligand.

Bis(2-ethylhexyl) phthalate occupies the active pocket of the PDE-5 enzyme, forming strong inhibitory bonds within its catalytic domain (Figure 2C). Moreover, it occupies the first ranked pose within the enzyme’s active pockets, as represented in a 2D depiction of the amino acids (Figure 2D). This compound is believed to effectively inhibit PDE-5, indicating its potential role in the aphrodisiac activity associated with *M. jalapa* root extract. In essence, these three compounds’ interactions with the PDE-5 enzyme through molecular docking highlight their inhibitory potential and compare them to established inhibitors, shedding light on their potential use in addressing conditions like erectile dysfunction.

The promising in silico aphrodisiac potentials of *M. jalapa* roots motivated us to further investigate and validate these findings through an in vivo study involving normal male rats. Through an in vivo experiment, our objective was to validate and interpret the possible aphrodisiac impacts of *M. jalapa* root extract within a living organism, providing more robust evidence of its therapeutic properties. Prior to conducting the in vivo assay, the safety of the plant extract was assessed through a limit test for toxicity. In this preliminary safety test, a total of 3 male rats were given 2000 mg/kg of the extract orally. This test was conducted to ensure the safety of the extract and establish appropriate dosage levels for the subsequent in vivo study. The results of the limit test indicated that the dosage level of 2000 mg/kg of the given plant extract was entirely safe for one-time oral use in rodent models. The results indicated that the given extract could not inflict any toxicity, morbidity, or mortality at the tested dose, establishing its safety for further experimentation in the subsequent in vivo study. However, a previously published study on 70% ethanolic extract conducted according to OECD Guideline 423 for acute toxicity study in mice found no sign of any toxic symptoms or any mortality [24].

**Figure 3:**
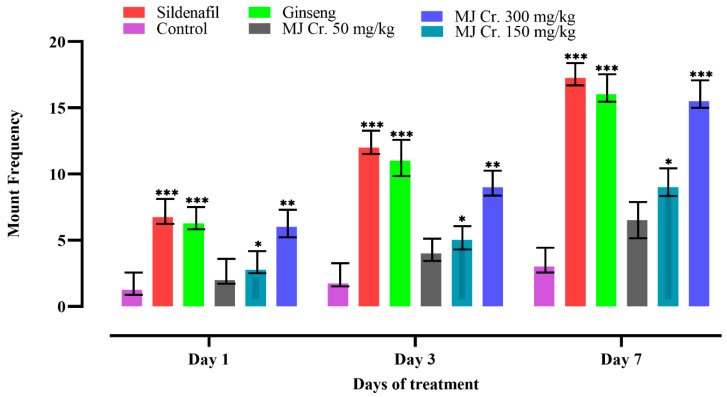
Effect of extract of *M. jalapa* roots on mount frequency in male albino rats. *** indicate (*p <* 0.01), ** (*p <* 0.03) and * (*p <* 0.05).

**Figure 4:**
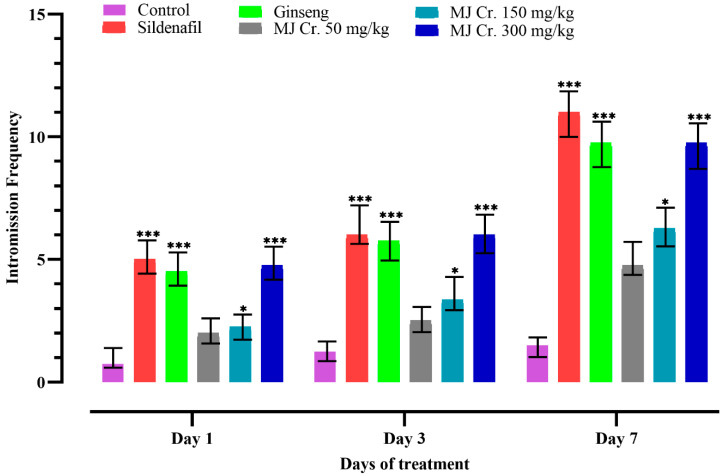
Effect of extract of *M. jalapa* roots on intromission frequency in male albino rats. *** indicate (*p <* 0.01), and * (*p <* 0.05).

**Figure 5:**
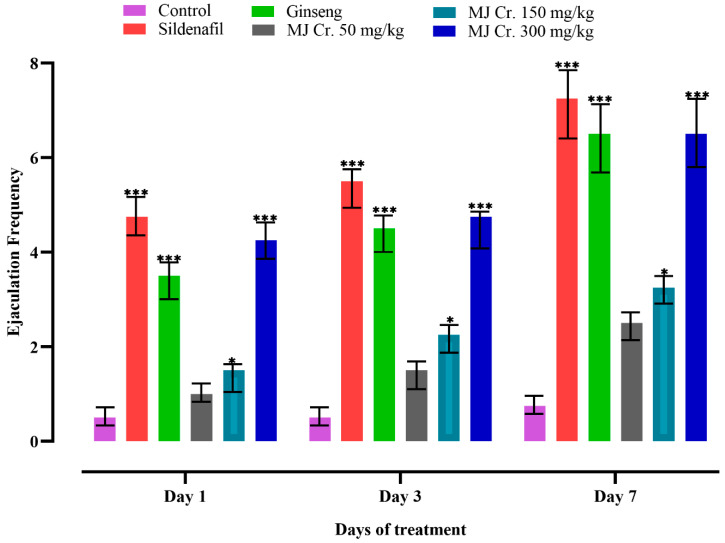
Effect of extract of *M. jalapa* roots on ejaculation frequency in male albino rats. *** indicate (*p <* 0.01) and * (*p <* 0.05).

Following the limit test, a comprehensive assessment of the aphrodisiac potential was conducted on male rats. Sexual parameters including mount frequency (MF), intromission frequency (IF), and ejaculation frequency (EF) were measured, which are indicative of libido, potency, and vigor. An increase in MF signifies enhanced sexual motivation, while an increase in IF suggests improved penile efficiency, erection, and orientation of penile muscle. Furthermore, an increase in EF indicates the aphrodisiac properties of the treatments. These sexual parameters served as important indicators in evaluating the aphrodisiac effects of the tested treatments on the male rats [20,25,26]. In the current study, the analysis of video recordings showed that rats administered with sildenafil, ginseng, and different doses of *M. jalapa* crude extract (MJ Cr.) exhibited increased MF, IF, and EF. On the other hand, the animals form the control group (received only distilled water) displayed lower MF, IF, and EF. Notably, the rats treated with the 300 mg/kg dose of MJ Cr. extract showed significant increases (*p* < 0.05) in all the mentioned parameters on the first, third, and seventh days of the treatment. Statistical analysis employing two-way ANOVA, further followed by Tukey’s test, verified that the period of treatment with MJ Cr. extract at a dosage of 300 mg/kg had a notable influence (*p* < 0.05) on the incidences of mounting, intromission, and ejaculation (Figure 3, Figure 4 and Figure 5).

**Figure 6:**
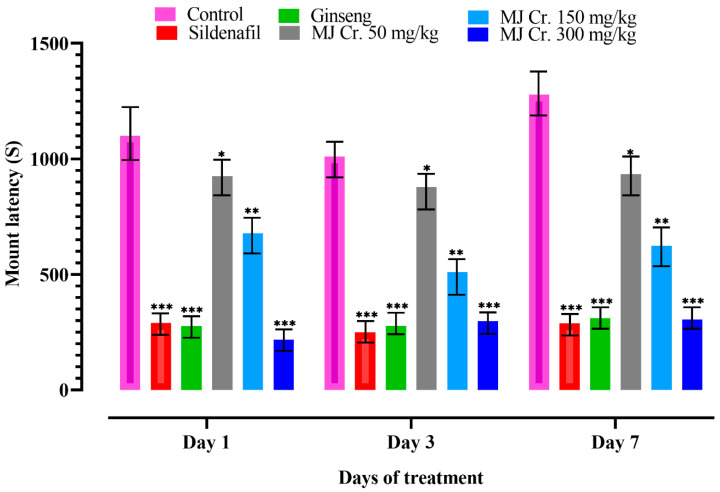
Effect of extract of *M. jalapa* roots on mount latency in male albino rats. *** indicate (*p* < 0.01), ** (*p* < 0.03), and * (*p* < 0.05).

**Figure 7:**
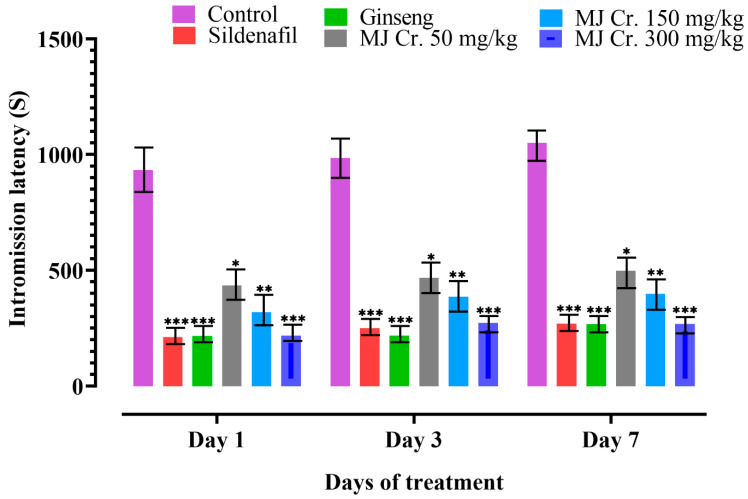
Effect of extract of *M. jalapa* roots on intromission latency in male albino rats. *** indicate (*p* < 0.01), ** (*p* < 0.03), and * (*p* < 0.05).

Parameters such as mount latency (ML) and intromission latency (IL) serve as significant indicators to assess sexual motivation and appetite in male rats. When ML and IL decrease, it indicates a shorter time interval between the introduction of a receptive female and the initiation of mounting or intromission behavior. This reduction in latency suggests an increased sexual drive and the potential aphrodisiac activity of the extract [18,27]. Therefore, in the present study, a decrease in ML and IL would indicate the efficacy of the extract in enhancing sexual motivation and performance in male rats. The male rats from the control group as well as the group receiving MJ Cr. 50 mg/kg extract exhibited ML and IL, indicating hesitation towards female rats. However, the rats treated with sildenafil, ginseng, MJ Cr. 300 mg/kg, and MJ Cr. 150 mg/kg extracts showed the lowest ML and IL, indicating the aphrodisiac properties of these treatments. Additionally, ML and IL decreased over time in rats treated with MJ Cr. 50 mg/kg, MJ Cr. 150 mg/kg, and MJ Cr. 300 mg/kg extracts, with the most significant reduction observed on days 3 and 7, respectively. The rats given an MJ Cr. 300 mg/kg extract displayed no significant variation (*p* < 0.05) from the rats treated with sildenafil as well as ginseng. Moreover, the ML and IL of rats treated with MJ Cr. 300 mg/kg extract were significantly lower (*p* < 0.05) than those in the control group. A statistical evaluation via two-way ANOVA, followed by Tukey’s test, confirmed that the treatment period using *M. jalapa* root extract exerted a substantial (*p* < 0.05) impact on both ML and IL (Figure 6 and Figure 7).

**Figure 8:**
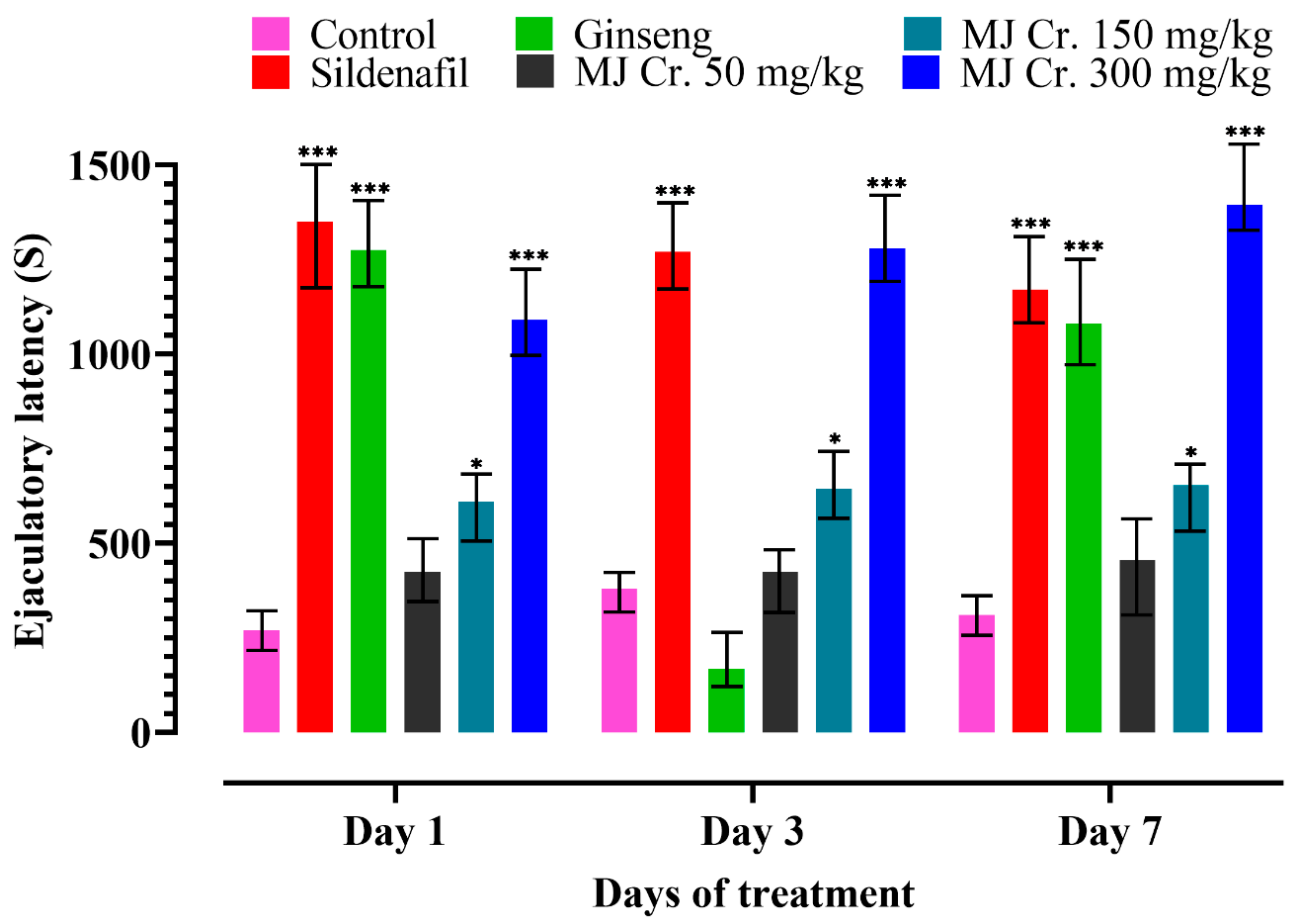
Effect of extract of *M. jalapa* roots on ejaculatory latency in male albino rats. *** indicate (*p <* 0.01) and * (*p <* 0.05).

An increase in EL indicates higher sexual motivation and direct sexual intercourse between male and female rats, bypassing mounting and intromission and leading to ejaculation. It demonstrates a significant improvement in the copulatory performance of both male as well as female rats [19,28]. Results of the present study also indicated that EL was the most prominently found in the rats treated with sildenafil, followed by those administered with MJ Cr. 300 mg/kg, and then those given ginseng. There was a significant (*p* < 0.05) difference in the ejaculatory latencies of the rats given MJ Cr. 300 mg/kg and MJ Cr. 150 mg/kg when compared to those in the control group. The least ejaculatory latencies were shown by the rats in the control group, followed by MJ Cr 50 mg/kg and MJ Cr 150 mg/kg, respectively. An analysis involving a two-way ANOVA, followed by Tukey’s test, suggested that the treatment durations did not significantly impact (*p* > 0.05) the ejaculatory latencies. This could likely be due to differing responses among the rats (Figure 8).

**Figure 9:**
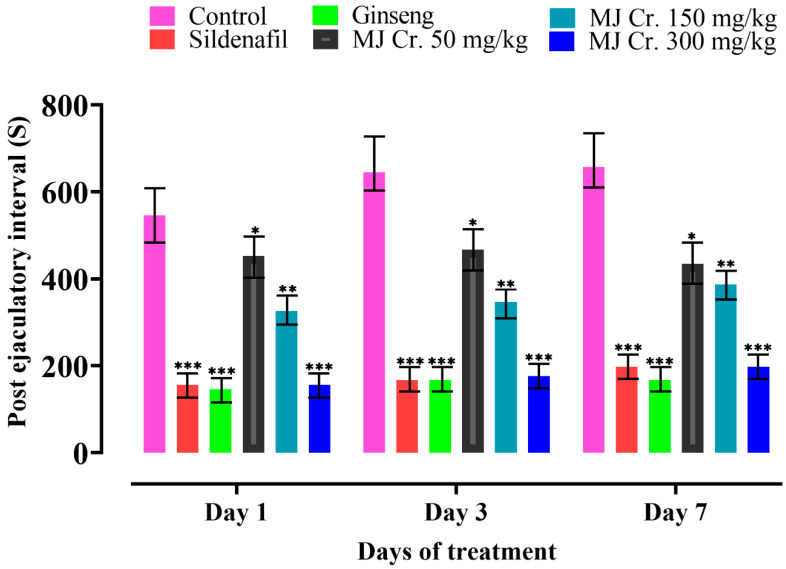
Effect of extract of *M. jalapa* roots on post-ejaculatory interval in male albino rats. *** indicate (*p* < 0.01), ** (*p* < 0.03), and * (*p* < 0.05).

The decrease in post-ejaculatory interval (PEI) observed in male rats indicates an increase in libido, potency, and a faster recovery rate from exhaustion during sexual intercourse. A shorter PEI suggests enhanced sexual vigor and a higher likelihood of engaging in subsequent sexual activity [29]. A notable reduction in PEI was observed in rats treated with MJ Cr. 300 mg/kg extract and followed by those administered with sildenafil and then ginseng, respectively. This indicates that these treatments resulted in shorter recovery times between ejaculations, reflecting better penile erections and improved copulation. The PEI of rats treated with MJ Cr. at various doses (300 mg/kg, 150 mg/kg, and 50 mg/kg) showed a significant difference (*p* < 0.05) compared to the rats in the control group. However, the statistical analysis, involving two-way ANOVA followed by Tukey’s test, revealed that the duration of treatment did not significantly affect PEI (Figure 9).

**Figure 10:**
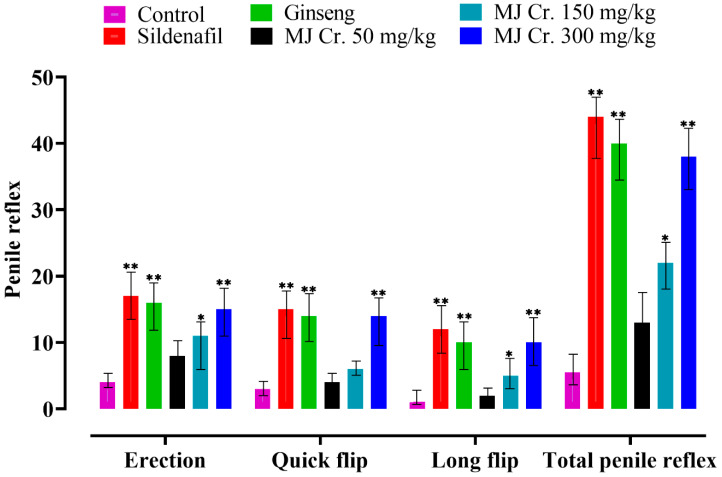
Effect of extract of *M. jalapa* roots penile reflexes of the male albino rats. ** indicate (*p* < 0.03) and * (*p* < 0.05).

Significant variations (*p* < 0.05) in erection and quick flip reflexes were observed in rats treated with sildenafil, ginseng, MJ Cr. 300 mg/kg, and MJ Cr. 150 mg/kg extracts when compared to the control group. Notably, the outcomes for erection and quick flip reflexes with the MJ Cr. 300 mg/kg extract were significantly similar to the results produced by the standard drug, sildenafil. These findings suggest that the MJ Cr. 300 mg/kg extract exhibited similar effects to sildenafil in promoting erection and quick flip reflexes, indicating its potential as an aphrodisiac agent. On the other hand, long flip reflexes exhibited by the rats administered with sildenafil, ginseng, MJ Cr. 300 mg/kg, as well as MJ Cr. 150 mg/kg were found higher and significantly different (*p* < 0.05) when compared to the control group. The control group and the rats were treated with MJ Cr. 50 mg/kg demonstrated the least improvement in long flips. In conclusion, the total penile reflexes were significantly improved in all the treated groups, except for the rats treated with MJ Cr. 50 mg/kg, which showed similar results to the control group. Among the treated groups, the highest total penile reflexes were observed in rats treated with sildenafil, followed by ginseng, MJ Cr. 300 mg/kg, and MJ Cr. 150 mg/kg. This suggests that these treatments have a positive impact on penile reflexes, indicating improved sexual performance (Figure 10).

Sexual hormones, including testosterone and luteinizing hormone, play a crucial role in sexual performance by enhancing libido and promoting erections. These hormones stimulate the release of neurotransmitters, such as dopamine, which, in turn, increase locomotor activity and contribute to improved copulatory and sexual performance. During mating and sexual encounters, the circulation of testosterone in the body enhances sexual desire, motivation, and overall sexual function [30,31]. As illustrated in Table 3, the rats treated with sildenafil, ginseng, and MJ Cr. 300 mg/kg exhibited significantly elevated (*p* < 0.05) levels of testosterone as well as luteinizing hormone in their blood samples. The other doses of *M. jalapa* root extract, such as MJ Cr. 150 and 50 mg/kg, also elevated the serum testosterone and luteinizing hormones but was not significant compared to the standard sildenafil and ginseng. Thereby, the observed improvement in the sexual performance of rats can be attributed to the overall effects of the *M. jalapa* root extract on hormonal regulation, leading to increased levels of testosterone and luteinizing hormone. In conclusion, sildenafil has been used as a benchmark to evaluate various factors, including serum testosterone and luteinizing hormone levels, in numerous in vivo studies exploring aphrodisiac effects. It has been repeatedly shown through extensive research that sildenafil administration increases serum levels of testosterone and luteinizing hormone. This has been true for both in vivo models and patient-based clinical trials. In ginseng-related studies that were previously published, it was noted that supplementing with ginseng extract increased levels of luteinizing hormone and testosterone. The ginseng plant’s root and rhizome extracts have long been revered for their traditional use as aphrodisiacs, aiding in the facilitation of penile erection and enhancing sexual activity. Additionally, taking ginseng supplements has been shown to have a beneficial effect on sperm quality and motility, underscoring its potential role in reproductive health. In our study, when *M. jalapa* extract was given at a dose of 300 mg/kg to the male rats, serum levels of testosterone and luteinizing hormone elevated in the same way that sildenafil and ginseng did. These results highlight the possibility that *M. jalapa* extract can affect the hormonal levels involved in reproductive function, potentially contributing to its aphrodisiac effects [32,33,34,35].

**Figure 11:**
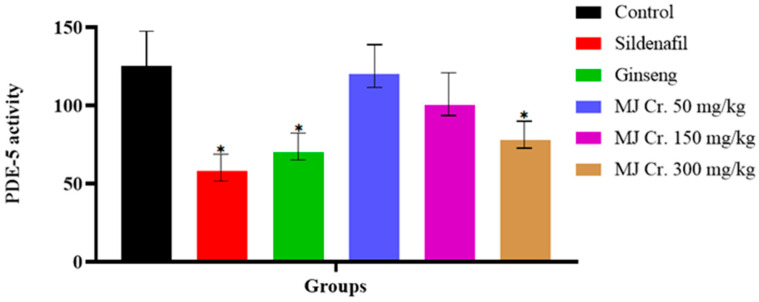
In vitro inhibitory effect of *M. jalapa* root extract on PDE-5 activity (* indicates *p* < 0.05).

The in silico analysis of *M. jalapa* root extract indicated its remarkable conformations and interactions with the target protein, PDE-5, and its in vivo aphrodisiac activities in normal rats further insighted us to investigate its enzyme inhibitory activity in vitro. In vitro enzyme inhibitory assay findings revealed that sildenafil exerted the most potent inhibitory action against PDE-5, followed by ginseng and then MJ Cr. 300 mg/kg, respectively. These inhibitory effects were notably different (*p* < 0.05) from that of the control group. Nevertheless, the inhibitory effects presented by MJ Cr. 150 and 50 mg/kg were comparatively mild and did not achieve statistical significance (Figure 11).

## 3. Materials and Methods

### 3.1. Collection of the Plant Material

The roots from *M. jalapa* L. roots were collected from district Buner, KPK, Pakistan in the month of September 2019, specifically from the coordinates; latitude: 34° 30′ 41.04″ N and longitude: 72° 29′ 2.04″ E. A taxonomy specialist, Dr. Abdul Nazir from the Environmental Sciences Department, COMSATS University, Islamabad, Abbottabad Campus, verified and identified the plant. A voucher specimen was assigned with the number CUHA-211 to authenticate the collected plant material. The collected plant specimen was preserved and stored in the University Herbarium for future reference and documentation. A total of 10 kg of *M. jalapa* roots were gathered, followed by the separation of roots from any dirt or foreign particles. The roots were subsequently dried in a shaded area. After being thoroughly dried, the roots were ground into a fine powder and kept in a sealed container before beginning the extraction process.

### 3.2. Extraction

For the extraction process, 500 g of *M. jalapa* L. root powder was taken and macerated in a mixture of 50% ethanol and 50% water, with a total volume of 2 L. The maceration process was carried out for a duration of 21 days to allow for efficient extraction of the bioactive compounds from the roots. The collected extract was subjected to evaporation utilizing a vacuum rotary evaporator with a set temperature of 40 °C, resulting in the formation of a semisolid residue. To further process the residue, freeze-drying was conducted for a period of 24 h. This process yielded the dried crude extract, which accounted for approximately 11% of the initial dry weight of the extract.

### 3.3. Phytochemicals Evaluation of the Hydroethanolic Extract

The hydroethanolic extract’s total phenolic and flavonoid contents were assessed via their respective assays [36,37] to determine their levels. Additionally, the analysis of metal and mineral contents was conducted using atomic absorption spectrophotometry (PerkinElmer, USA) in accordance with a method already described [38]. Furthermore, the metabolic makeup of the extract was examined using techniques such as gas chromatography and mass spectrometry (GC-MS) with a PerkinElmer Clarus 600 Gas Chromatograph by the method already established [39]. The GC-MS spectra were obtained using Turbo Mass version 5.4.0 software and compared with standard compounds using the NIST library database, enabling the recognition and comparative analysis of the compounds contained in the extract.

### 3.4. Molecular Docking Study

Docking studies were conducted using the three compounds identified in the GC-MS analysis of the *M. jalapa* extract. These docking studies aimed to predict the most probable mechanism by which these compounds may exert their biological effects. For the in silico studies, various software tools were utilized. Autodock Vina version 18 was employed for molecular docking analysis to investigate the binding interactions between the ligands and the target enzyme (PDE-5). Pymol visualizer version 2.5 was used to study the ranking of the ligands in the catalytic domain of the enzyme PDE-5. Discovery Studio version 21.0.1 was employed to analyze the different catalytic pockets of the enzyme and examine the bonding between the ligands derived from the plant extract and the enzyme. The enzyme PDE-5 was procured from the data bank website, www.rcsb.org, which serves as a reliable source for retrieving structural information on biomolecules [40].

### 3.5. Experimental Animals

The experiments were conducted on normal male Wistar rats, which were bred in the animal housing unit of COMSATS University Islamabad, Abbottabad campus, Pakistan. The rats were housed in clean metallic cages and provided with standard food pellets and tap water. They were kept under standard environmental conditions, including a 12 h light/dark cycle and a temperature maintained at 24 ± 2 °C. This experimental procedure received official approval from the Research Ethical Committee on the Care and Use of Laboratory Animals of the Department of Pharmacy, COMSATS University Islamabad, Abbottabad campus, Pakistan (REF: PHM-Eth/CF-M04/11-24), in accordance with the guidelines outlined in the NIH guidelines for the handling and usage of laboratory animals (NIH Publication No. 80–23; revised 1978).

### 3.6. Experimental Design

To ensure the safety of the extract before conducting an in vivo aphrodisiac activity, a limit test for the toxicity was performed. The test involved administering the extract to three male rats, each weighing 270 ± 5 g, at a dose of 2000 mg/kg of body weight. The purpose of this test was to evaluate any potential adverse effects or toxicity associated with the given extract at this dosage. The rats were closely monitored for any apparent signs of toxicity or adverse reactions throughout the period of observation.

The aphrodisiac model was designed using 36 male (weighing 270 ± 5 g) and 36 female (weighing 250 ± 4) rats, which were subsequently assigned into six groups on a random basis. Each group consisted of six male and six female rats (*n* =12). The activity of the extract was compared with two standard drugs, sildenafil (Pfizer*^®^*, New York, NY, USA) and ginseng (Korea Ginseng Corp^®^, Daejon, Republic of Korea). Estradiol benzoate (10 mg/kg of the body weight) and progesterone (0.5 mg/100 g of the body weight) were successively administered under the skin (subcutaneously) at 48 h and 4 h, respectively, to the female rats to make them receptive for pairing before the start of the experiment [41]. In the current study, the male rats were divided into different treatment groups as follows:

Group 1: Rats receiving only vehicle (distilled water);
Group 2: Rats receiving 50 mg/kg of sildenafil;
Group 3: Rats receiving 300 mg/kg of ginseng;
Group 4: Rats receiving 50 mg/kg of *M. jalapa* crude extract (MJ Cr.);
Group 5: Rats receiving 150 mg/kg of *M. jalapa* crude extract (MJ Cr.);
Group 6: Rats receiving 300 mg/kg of *M. jalapa* crude extract (MJ Cr.).

These treatments were administered to the rats at 19:00 on the first, third, and seventh days of the study. After a 30 min interval, the rats were paired with female rats for mating. Sexual parameters such as mount frequency (MF), intromission frequency (IF), ejaculatory frequency (EF), mount latency (ML), intromission latency (IL), ejaculatory latency (EL), and post-ejaculatory interval (PEI) were recorded by analyzing video recordings taken from 19:30 to 22:30, totaling three hours. The methodology followed in the present study has already been described previously [27].

### 3.7. Penile Reflexes Test

On the 8th day of the experiment, after a 30 min interval from the administration samples and standards, the male rats were subjected to a penile reflexes test. The rats were placed in a glass cylinder on their back, and the preputial sheath was gently pushed behind the glands for a duration of 15 min. The frequency of penile reflexes, including erection (E), quick flips (QF), long flips (LF), and total penile reflexes (TPR), was recorded and calculated. These penile reflexes serve as indicators of the male rats’ sexual response and were used to evaluate the impact of the extract and standard drugs related to sexual performance [27].

### 3.8. Measurement of the Levels of Testosterone and Luteinizing Hormone in Serum

At the end of the experiment, the male rats were humanely sacrificed, and the blood samples were collected via cardiac puncture to estimate the levels of luteinizing hormone (LH) and testosterone using respective assay kits (Monobind Inc., based in Lake Forest, CA, USA). The levels of LH and testosterone in the serum were estimated according to the guidelines given by the assay kit manufacturers. For this purpose, the assays were performed using microplate immune enzymometric (EMA/ELISA) procedures. The serum hormone concentrations were determined by interpolating the values obtained from their individual calibration curves and the chemistry analyzer used for this analysis was calibrated and validated for use with rat sera, ensuring accurate measurement of LH and testosterone levels.

### 3.9. In Vitro Phosphodiesterase-5 (PDE-5) Inhibitory Assay

The PDE-Glo phosphodiesterase assay kit was utilized to quantify the activity of phosphodiesterase (PDE-5). Tissue from the penis was extracted and then mixed with a solution of RIPA lysate buffer. This mixture was then centrifuged at 4 °C for a quarter of an hour. The clear liquid, or supernatant, that was obtained post-centrifugation was then processed according to the kit’s recommended procedure to determine PDE-5 activity. This involved allowing the penis tissue to incubate with the cyclic guanosine monophosphate (cGMP) substrate to commence the phosphodiesterase reaction. Following this, a phosphodiesterase (PDE) detection solution comprising adenosine triphosphate (ATP) and protein kinase A (PKA) was subjected to a treatment with the PDE-Glo termination buffer (PKA). The ATP consumption in this reaction, which correlates to the level of cGMP, was evaluated using a luciferase-dependent Kinase–Glo reagent. After an incubation period of 10 min at ambient temperature, the sample’s optical density was measured utilizing a SpectraMax L microplate luminometer (MDS AT (US) Inc., Stuart, FL, USA). The resultant data was displayed as a percentage in relation to the control and was documented in the form of relative light units (RLUs) [42].

### 3.10. Statistical Analyses

The data obtained from the study were analyzed using a two-way analysis of variance (ANOVA) followed by Tukey’s test. GraphPad Prism 8 software, developed by GraphPad Software Inc., in San Diego, CA, USA, was utilized for the statistical analysis. The data were expressed as the mean of the six replicate determinations ± SD, and the significance of the data was declared at the level of *p* < 0.05.

## 4. Conclusions

The hydroethanolic extract of *M. jalapa* roots is a rich source of biologically active phytochemicals (phenols and flavonoids), minerals, and metals. It exhibited promising in silico and in vitro aphrodisiac potential by effectively inhibiting the activity of PDE-5. The extract also demonstrated high safety in rodent models, even at doses as high as 2000 mg/kg. The in silico and in vitro aphrodisiac potentials of *M. jalapa* root extract were successfully validated through in vivo studies in normal male rats. The extract enhanced the libido, vigor, sexual motivation, and copulatory behavior of male rats, as evidenced by higher frequencies of mount, intromission, and ejaculation. It also led to decreased latencies of mounting and intromission. Furthermore, the extract increased the levels of serum testosterone and luteinizing hormone, further supporting its aphrodisiac effects. The findings of this study provide strong support for the aphrodisiac activity of *M. jalapa* root extract and its potential use in the treatment of ED or premature ejaculation. By employing alternative green extraction methods, a standardized herbal extract of *M. jalapa* roots can be developed, offering a promising therapeutic choice for individuals with ED. The limitation of the current study includes histopathological examination of the rat testes and penis, which will be performed in future studies based on *M. jalapa* root extract dosage form development.

## Figures and Tables

**Table 1:** Analysis of the metal and mineral content in *M. jalapa* root extract.

S. NO.	Metals and Minerals	Concentration (mg/L)
1	Magnesium (Mg)	14.17
2	Cobalt (Co)	0.130
3	Arsenic (As)	0
4	Cadmium (Cd)	0.011
5	Calcium (Ca)	27.91
6	Iron (Fe)	0.026
7	Zinc (Zn)	40.24
8	Chromium (Cr)	0.031
9	Sodium (Na)	5.761
10	Potassium (K)	39.70
11	Lead (Pb)	1.306

**Table 2:** Compounds identified via GC-MS analysis of the *M. jalapa* root extract.

Peak No	Compound	Mol. wt.	M. Formula	Ret. Time	Peak Area (%)
**1**	2,6-Octadien-1-ol, 3,7-dimethyl-, (z)-	154	C_10_H_18_O	5.424	0.488
**2**	Triethanolamine	149	C_6_H_15_O_3_N	9.276	3.03
**3**	Butanoic acid, 3,7-dimethyl-2,6-octadienyl ester, (e)-	224	C_14_H_24_O_2_	12.21	8.35
**4**	Beta. -d-glucopyranoside, methyl	194	C_7_H_14_O_6_	14.42	0.25
**5**	*N*-hexadecanoic acid	256	C_16_H_32_O_2_	25.183	1.93
**6**	Heptadecanoic acid, ethyl ester	298	C_19_H_38_O_2_	25.873	0.38
**7**	9-benzyl-2,6-dichloro-9H-purine	279	C_12_H_8_C_l2_N_4_	31.020	6.5
**8**	N-propyl 11-octadecenoate	324	C_21_H_40_O_2_	31.260	7.1
**9**	1-Nonylcycloheptane	224	C_16_H_32_	31.84	0.69
**10**	(1*S*,15*S*)-Bicyclo [13.1.0] hexadecan-2-one	236	C_16_H_28_O	34.60	0.87
**11**	Hexanedioic acid, bis(2-ethylhexyl) ester	370	C_22_H_42_O_4_	38.593	4.10
**12**	Bis(2-ethylhexyl) phthalate	390	C_24_H_38_O_4_	41.609	0.15

**Table 3:** Effect of *M. jalapa* root extract on luteinizing and testosterone hormone levels in male rats.

S/NO	Group	LH (mIU/mL)	Testosterone (ng/mL)
1	Control	4 mIU/mL	7.5 ng/mL
2	Sildenafil 50 mg/kg	6 mIU/mL **	9.5 ng/mL **
3	Ginseng 300 mg/kg	5.8 mIU/mL **	8.9 ng/mL **
4	MJ 50 mg/kg	4.7 mIU/mL	7.7 ng/mL
5	MJ 150 mg/kg	4.9 mIU/mL	7.9 ng/mL
6	MJ 300 mg/kg	5.8 mIU/mL **	9 ng/mL **

LH: Luteinizing hormone; ** indicates *p* < 0.03.

## Data Availability

The data presented in this study are available in the article.

## Sample Availability

Samples of the compound MJ Cr. are available from the authors on demand.
